# Strategies to Overcome Antimicrobial Resistance (AMR) Making Use of Non-Essential Target Inhibitors: A Review

**DOI:** 10.3390/ijms20235844

**Published:** 2019-11-21

**Authors:** Giannamaria Annunziato

**Affiliations:** Probes for Targets Group (P4T group), Department of food and Drug, University of Parma, 43124 Parma, Italy; giannamaria.annunziato@unipr.it

**Keywords:** antibiotic resistance, non-essential targets, antibiotic adjuvant therapies, virulence factors, combination therapy, beta-lactamases inhibitors, efflux pump inhibitors, membrane permeabilizers

## Abstract

Antibiotics have always been considered as one of the most relevant discoveries of the twentieth century. Unfortunately, the dawn of the antibiotic era has sadly corresponded to the rise of the phenomenon of antimicrobial resistance (AMR), which is a natural process whereby microbes evolve in such a way to withstand the action of drugs. In this context, the identification of new potential antimicrobial targets and/or the identification of new chemical entities as antimicrobial drugs are in great demand. To date, among the many possible approaches used to deal with antibiotic resistance is the use of antibiotic adjuvants that hit bacterial non-essential targets. In this review, the author focuses on the discovery of antibiotic adjuvants and on new tools to study and reduce the prevalence of resistant bacterial infections.

## 1. Introduction

Antibiotics have always been considered as one of the most relevant discoveries of the twentieth century and their widespread use has revolutionized healthcare since their introduction [1]. The dawn of the modern antibiotic era is associated with the names of Paul Ehrlich and Alexander Fleming. Paul Ehrlich is credited with the concept of the so-called “magic bullet”, based on target selectivity. Ehrlich asserted that it was possible to obtain chemical compounds able to exert their pharmacological action exclusively on the parasite. On the other hand, Fleming is to be credited for the serendipitous discovery of penicillin, later to be claimed as the miracle drug of the twentieth century. Although the spread of the antibiotics formally starts with the antibiotic era, in the pre-antibiotic era, numerous remedies belonging to the traditional medicine have constantly put microorganisms under selective pressure. Antimicrobial activity seems to be present in a number of herbs and plants and the discovery of active components from natural sources has not only contributed to build the antibacterial arsenal, but they still keep fueling the pipeline of antimicrobials used by mainstream medicine. In the past 200 years, empirical sciences and serendipity have come together to bring over the current state of knowledge about antimicrobial drugs [2]. Between 1950 and 1970, in the so-called “golden age” of antibacterial drugs discovery, the empirical screening of microbial natural products’ fermentation provided the majority of antibacterial classes currently used for the treatment of infections [3].

Over the last 30 years, antibacterial drug discovery has faced a discovery gap, and no new classes of antibacterials were introduced in the market until 2000, when linezolid, belonging to the class of oxazolidinones, was approved [4,5].

**Figure 1:**
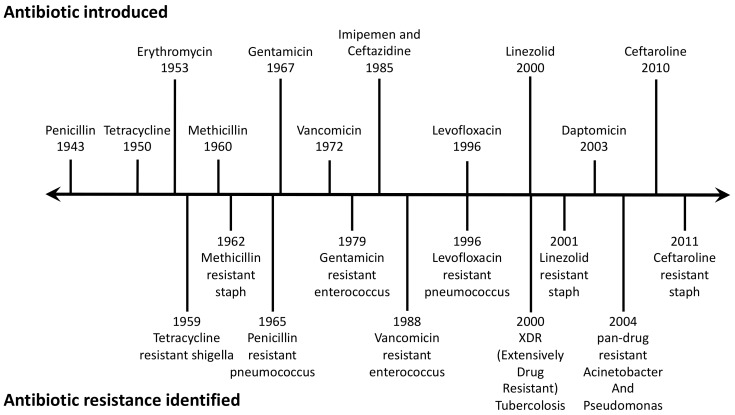
Antibiotic resistance pipeline. On the top, antibiotic introduced and, on the bottom, antibiotic resistance identified.

Unfortunately, the dawn of the antibiotic era has sadly corresponded to the rise of the phenomenon of antimicrobial resistance. It is widely accepted that, while the use of antimicrobial agents has led to the control and even eradication of infectious diseases, their misuse and/or overuse has led to the development of resistant strains. Based on his early observations, in 1954, Fleming had predicted that the inconsiderate use of this discovery could lead to the selection and propagation of selected mutants of bacteria resistant to antibiotics. Indeed, alarming signals of developing resistance were observed after only a few years of the golden age of antimicrobials [6] (Figure 1).

Antimicrobial resistance (AMR) is a natural phenomenon whereby bacteria evolve in such a way to withstand the action of drugs, making them apparently ineffective. The pressure that antimicrobials put on the pathogens is responsible for the selection of resistant strains [7]. Although AMR is a naturally occurring process, and while in the past decades it was believed to be under control, today it is considered a threat for global health and the expectations for the future are not encouraging [8]. There are three main causes of AMR. The first cause is considered to be the use of antimicrobials, which has strongly increased in the last few decades. The second cause lies in the fact that in most cases, the patients are not able to follow the therapy instructions properly. The third cause is due to the fact that within a given class of antimicrobials, there are very limited numbers of new drugs under development to replace those rendered ineffective by rising drug resistance. Furthermore, research on antibiotic pharmacokinetic (PK) and pharmacodynamic (PD) properties that maximize the probability of a successful outcome is needed. Indeed, it is possible that the patient is exposed to sub-therapeutical concentration of the drug for reasons that could be ascribed to an inappropriate dosing, alteration in renal and/or hepatic function, variation in protein binding, or volume of distribution. Resistance can be divided into two groups: intrinsic resistance or acquired resistance. While intrinsic resistant bacteria refer to microorganisms that do not have the target for the antibiotic compound, acquired resistance refers to originally susceptible bacteria that acquire a mechanism that allow them to evade the action of the antibacterial [9]. Acquired resistance can arise through casual mutations or through acquisition of external genetic material. Horizontal gene exchange arises through plasmids or through transposons. Plasmids are a small molecule of DNA on which genes are assembled and reorganized. The multitude of potentially useful genes allow a bacterial strain to expand its area of viability into niches that were previously too harsh or too hazardous to survive. In this way, plasmids are allowed to transfer genetic information to other Gram-positive or Gram-negative bacteria.

**Figure 2:**
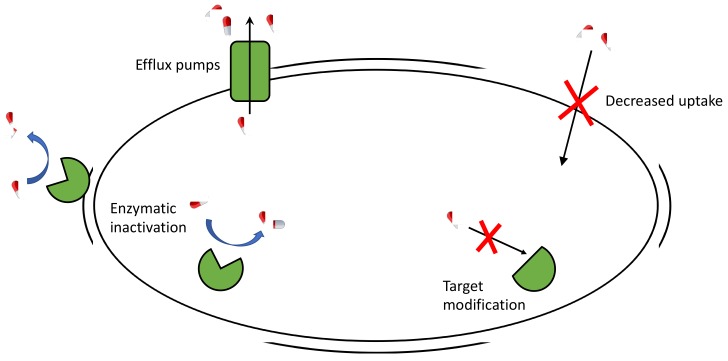
Cartoon representation of different mechanisms of antibiotic resistance. Antibiotics as red and white pills, target proteins in green.

There are four mechanisms through which bacteria become resistance to antibiotics [10] (Figure 2):

1. Enzymatic inactivation: An existing bacterial enzyme is modified to interact with an antibiotic in order to make them inactive towards bacteria. It is due to the transfer of the antibiotic resistance gene carried on plasmids. The most significant examples are beta-lactamase enzymes, which hydrolyze beta-lactams (penicillins, cephalosporins).
2. Drug extrusion by efflux pumps: These proteins, which are able to extrude a wide variety of compounds (including antibiotics) out of the cell, are overexpressed by the bacteria to extrude the antibiotic. This is an important mechanism of resistance in *P. aeruginosa* and *Acinetobacter* spp.
3. Decreased uptake by changes in the outer membrane permeability or by presence of porins: These variations interfere with the entrance of antibiotics.
4. Modification of the drug target: These changes impede the binding of the antibiotic and limit its potency.

An extensive list of AMR bacteria was recently published by the World Health Organization (WHO) on the fact sheet of 27 February 2017. Pathogens are classified as critical, high, and medium, and this classification is based on mortality, level of resistance, and treatability. The situation is highly critical in infections caused by the Gram-negative ESKAPE: *Enterecoccus faecium*, *Staphylococcus aureus*, *Klebsiella pneumoniae*, *Acinetobacter baumanii*, *Pseudomonas aeruginosa*, and *Enterobacter*. These bacteria are resistant to carbapenemes, that are considered as last resort antibiotics. *Mycobacterium tuberculosis*, the causative agent of tuberculosis, that has been added to this list, is considered as a top global health priority. They might cause serious and deadly infections such as bloodstream infections and pneumonia infections.

These bacteria are resistant to many antibiotic drugs of the current pipeline, including carbapenems and third generation cephalosporins—the most efficacious antibiotics for treating multi-drug-resistant bacteria. That resistance is associated with high mortality due to the absence of new antimicrobials in the pipeline. This priority list of pathogens is to be considered as an indicator for the prioritization of incentives and funding for Research and Development (R&D) aimed at discovering new effective antibacterial drugs [11].

Presently, multi-drug-resistant (MDR) bacteria cause about 25,000 deaths in Europe each year [12] and have an economic burden of about €1.5 billion annually. In the USA, the scenario is similar, with MDR bacteria causing the death of 23,000 people among the 2 million infected annually. For this reason, AMR is considered by WHO as one of the three most important public health threats of the twenty first century [13,14].

As the protection of antibiotic therapy is diminishing, it is important to preserve its efficacy and to reinforce our current arsenal of antibacterial drugs. In addition, an improvement in antibiotic compliance [15] is important. Therefore, improvements can be made by (i) providing new drugs to replace those made obsolete or (ii) prolonging the lifetime of current antibiotics.

The first objective is hard to reach without significant government financing, and in the last few years, we have experienced a cut in investment by pharmaceutical companies for the discovery of new antibiotic drugs [16]. The reasons for this reduction in funding are both economic and scientific obstacles. From an economic point of view, new antibiotics have a poor profitable return on investment compared to drugs targeting chronic diseases, due to the fact that antibiotic therapies are shorter in duration and are often curative. Furthermore, antibiotics will always become ineffective toward resistant bacteria, and this fact reduces profitability [17]. There are also scientific challenges, they consist in the fact that only two new antibiotic classes have been introduced to the clinic since the “golden age”. The antibiotics that are in development are derivatives of already approved drugs, against which, many bacteria already possess resistance mechanisms [18].

Taken together, it is crucial to look for new and more potent anti-infective drugs and to develop antibiotics with different scaffolds and with new mechanisms of action. Large efforts are being made, particularly in academia, to investigate the potential of an exploited essential process in bacteria and to develop new molecules that target them, as well as to study the biochemical basis of these targets. In addition, promising success has been achieved with those approaches that are focused on targeting non-essential pathways in order to minimize the impact of resistance to antibiotics by the use of an antibiotic “adjuvant” in combination with an antibiotic [19].

It has already been proven that synergy and drug combinations are a winning strategy in fighting MDR bacteria and might help in protecting the already existing drugs through the use of antibiotic adjuvants. The most known and successful example is the combination of Amoxicillin and clavulanic acid. Amoxicillin is a potent beta-lactam that is inactivated by beta-lactamases, and clavulanic acid is an inhibitor of beta-lactamases endowed with a weak antibacterial activity. This association, of an antibiotic molecule together with an antibiotic adjuvant, has led to Augmentin, which was the best-selling drug in 2001. Antibiotic adjuvants are molecules with weak or absent antimicrobial activity but that are able to enhance the activity of antibiotics, minimizing or blocking resistance. Antibiotic adjuvants can also suppress intrinsic resistance and this has led to an expansion of the activity spectrum of antibiotics. As a matter of fact, in the literature, there are examples reporting the use of Gram-positive selective antibiotics to treat infections caused by Gram-negative. This is also a good strategy with the antibiotics for which the toxicity is a problem, such as colistin. In this case, antibiotic adjuvants that increase bacterial susceptibility to antibiotics, would allow them to be efficacious at lower doses, thereby mitigating potential side effects [20].

To date, three main types of antibiotic adjuvants have been developed to block the aforementioned mechanisms of antibiotic resistance: (a) beta-lactamase inhibitors, (b) efflux pumps inhibitors, and (c) outer membrane permeabilizers. In Table 1 are reported a list of antibiotic adjuvants that are discussed in this review.

In addition, to enhance the antimicrobial action of drugs, new potential inhibitors are designed to target pathogenicity, in other words, the capacity of the bacterium to cause an infection [21]. This approach involves the identification of proteins, genes, and other biomacromolecules, responsible for bacterial virulence, whose inhibition would reduce bacteria fitness, making them more susceptible to the immune system’s attack and to antibiotics. Indeed, it has been proposed that such targets, although not critical for survival per se, are less likely to generate mutations. Besides, to expand drug targets, small molecules that target these “non-essential” genes can be combined with pre-existing antibiotics [22].

In this review, the author provides an overview of the antibiotic adjuvant strategies.

## 2. Targeting Efflux Pumps

Efflux pumps (EPs) are among the most well-known examples of bacterial mechanisms that confer cross-resistance to different antibiotics [23]. This type of resistance mechanism involves the antibiotics that exert their antibacterial activity inside the bacterial cell, in particular, macrolides, fluoroquinolones, and tetracyclines [24]. The efflux systems are able to actively extrude conventional antibiotics, leading to an increase of their minimum inhibitory concentration (MIC) or, in some cases, the loss of their antimicrobial activity. These systems are not only able to extrude antibiotics but also non-antibiotic substrates such as detergents and heavy metals, among others [25,26,27]. These membrane-spanning proteins are ubiquitously expressed in almost all living organisms including humans. Depending on their sequence similarity, substrate specificity, structural fold, and energy source, efflux pumps are classified in five families, namely: (i) the adenosine triphosphate (ATP)-binding cassette (ABC) superfamily, (ii) the resistance-nodulation-division (RND) family, (iii) the small multi-drug resistance (SMR) family, (iv) the major facilitator superfamily (MFS), and (v) the multi-drug and toxic compound extrusion (MATE) family [28].

MFS, ABC, SMR, and MATE families are expressed in both Gram-positive and Gram-negative bacteria, while the RND one is characteristic of the Gram-negative bacteria [29]. The ABC family utilizes ATP hydrolysis as an energy source to actively extrude the substrate, while the other ones use the proton gradient as an energy source.

It is widely believed that they usually confer a moderate level of resistance and the consequences of antibiotic active efflux can be summarized as follows:

1. Apparent poor antibiotics permeability: in some bacteria, it can be related to the expression of efflux pumps, which confers a resistance [23]. The best example is *Pseudomonas aeruginosa*, in which the knocking-down of the *mexB* gene produces mutants which are more susceptible to different classes of antibiotic (e.g., chloramphenicol, fluoroquinolones, tetracyclines, or beta-lactams) [30].
2. Cross-resistance to unrelated antibiotic classes: Cross-resistance comprises evolutionary events of the adaptation of antibiotics, or any other antimicrobial drug, which decreases the organism’s sensitivity to multiple drugs. This can be due, generally, to a high exposure to a given antibiotic.
3. Wide spectrum resistance can be observed in bacteria in which active efflux functions synergistically with other mechanisms of resistance, for example, in the *E. coli* strain that expresses both beta-lactamases and efflux pumps, and which is also insensitive to beta-lactams [31]. Thus, it has been found that the combination of these two mechanisms of resistance (efflux pumps and beta-lactamases) increases the level of resistance to quinolones [32].
4. Mutations can be favored in bacteria overexpressing efflux pumps. Indeed, in that condition, antibiotic targets become exposed to subinhibitory concentrations and can mutate to inhibit the effect of antibiotics [33], eventually conferring high-level resistance.

The active efflux of antibiotics was described for the first time 30 years ago. At that time, the presence of plasmid-encoded proteins able to extrude tetracycline and confer resistance to this antibiotic in *E. coli* [34] was studied by McMurry and colleagues. Since then, several classes of efflux pumps, both in Gram-positive and Gram-negative pathogens, have been characterized.

Nowadays, efflux pumps can be considered as potential antibacterial targets, due to their role in antibiotic resistance, and the development of inhibitors could improve the therapeutic arsenal against resistant pathogens. In the context of antibiotic combination therapy, efflux pumps are different from other mechanisms of resistance (such as beta-lactamases) that work on a specific family of antibiotics. Indeed, a single efflux pump can extrude a wide range of different families of antibiotics and, for this reason, their inhibition will increase the bacterial susceptibility and their combination could work with several antimicrobials.

There are several ways for inhibiting efflux pumps: (i) interfering with efflux gene expression, (ii) adding functional groups to the drug substrate to hamper recognition, (iii) interfering with the assembly of channel proteins, (iv) developing small-molecules as substrate analogues able to block the efflux pump activity, or (v) able to disjoin the energy transfer mechanism of the pump, or (vi) able to obstruct the channel [35,36].

Therefore, it is possible to corroborate that inhibition of efflux might lead to a variety of positive effects: (i) increasing the activity of the antibacterial drugs subject to efflux, (ii) keeping the concentration of the drug at the therapeutic dose, and (iii) shortening the duration of treatment by reducing multi-drug tolerance [37,38].

The most widely exploited strategy is the development of efflux pump inhibitors (EPIs), which are intended for combination therapy with specific antibiotics.

EPIs are small molecules that are able to bind efflux pumps and block their extrusion activity. EPIs, usually, do not have intrinsic antibacterial activity. For this reason, these compounds are further tested for synergy with different concentrations of antibiotics against a single concentration of inhibitor in bacterial strains containing efflux pumps. Inhibitors showing an eight-fold or more synergistic reduction in MIC are further evaluated by using the fractional inhibitory concentration (FIC) method [39].

**Figure 3:**
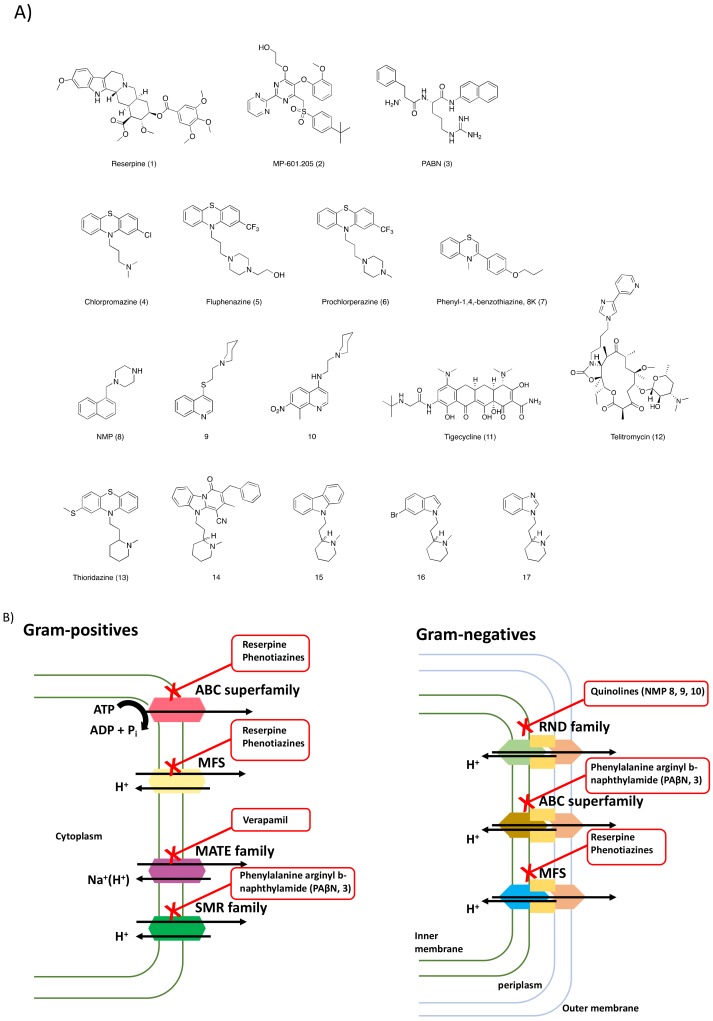
(**A**) Chemical structures of the efflux pump inhibitors (EPIs) discussed in this review and (**B**) efflux pumps expressed in Gram-positives and Gram-negatives bacteria and their respectively inhibitors.

One of the inconveniences in targeting these pumps is linked to the variety of physiological functions they are involved in, which can cause related toxicities when they are blocked, especially for the EPIs derived for a drug repurposing approach. Indeed, the main issue in such combination therapy is related to the need to use EPIs at high doses, underlying potential on- and off-targets side effects. In this context, research is focused on finding compounds that selectively inhibit pumps working only in prokaryotic cells [40]. Given this need, a large number of studies have been carried out to identify the substrates and inhibitors of these pumps. The first EPIs were serendipitously discovered from existing drugs. The most popular one is reserpine (1, Figure 3), that was shown to inhibit multi-drug transporters like NorA [41], increasing the intracellular concentration of fluoroquinolones, thus lowering MICs.

Similar effects were described with the phenothiazines, calcium channel antagonists, selective inhibitors of serotonin reuptake, or proton pump inhibitors. Derivatives lacking the pharmacological activity of the cognate compound are now developed, as described for inhibitors of serotonin reuptake or of omeprazole. To date, the only documented inhibitor is currently MP-601 (2, Figure 3) administered as an aerosol in patients with ventilator-associated pneumonia or cystic fibrosis [40,42].

Among the many strategies by which it is possible to inhibit efflux pumps’ activity, there are compounds able to compete with the antibiotics for their extrusion. The EPI lead compound is a dipeptide amide, named phenylalanine-arginine-β-naphthylamide (PaβN, 3, Figure 3), which inhibits several but not all RND efflux pumps. It has been found to improve or restore the activity of different classes of antibiotics, including 4-fluoroquinolones, macrolides, and chloramphenicol in a wide range of pathogens [43]. This molecule shows a competitive mechanism of action consisting of its binding to the targeted transporter at the same site used by the efflux pump to bind the antibiotic that it extrudes [44]. However, this molecule and its derivatives are too toxic to be used in therapy [45]. Other molecules with efflux pumps’ inhibition activity are the phenothiazine derivatives (4–7, Figure 3), and there have been many efforts to optimize them for therapeutic use. It was reported that phenothiazines improved the activity of different classes of antibiotics, including erythromycin, levofloxacin, and azithromycin. The mechanism of action of this class of EPIs is linked to interference with the proton gradient at the inner membrane of the bacteria [46]. Chlorpromazine also inhibits AcrB in salmonella enterica, but not by directly inhibiting efflux pumps but by exerting their synergistic activity by modulating the expression of the *acrB* gene [47].

Quinolines have shown inhibition of antibiotic efflux in isolates of the multi-drug-resistant bacteria. Indeed, it was shown that a number of quinoline derivatives are capable of potentiating activity of antibiotics through the inactivation of the AcrAB-ToIC (RND family) efflux transporters [48]. Studies on this class of compound showed that they were able to act synergistically with tetracycline, norfloxacin, and chloramphenicol in isolates of Gram-negatives, *K. pneumoniae* and *E. aerogenes* [49].

Another group of EPIs are the *N*-heterocyclic compounds, in particular the arylpiperazine derivatives (8–10, Figure 3), that have shown activity against both AcrAB and AcrEF efflux pumps in *E. coli* [50]. The leading compound of this class, 1-(1-Naphtylmethyl)-piperazine (NMP, 8, Figure 3), has shown very good EPI activity. Its action consists of reversing drug resistance in *E. coli* clinical isolates, making them susceptible to fluoroquinolones [51]. Regarding the mechanism of action of this class of EPIs, it has been suggested that NMP inhibits the AcrB efflux pump by interfering with its functional assembly that plays an important role in the extrusion of several substrates. The biggest disadvantage in using arylpiperazines is due to their relatively low potency and their similarity with serotonin agonists [40].

Alternatively, the extrusion of the antibiotic can be hampered, modifying its structure in order to reduce the efflux pump’s affinity. In this context, in the tetracycline and macrolide class of derivatives, the new compounds belonging to the glycylglycine and ketolide classes differ from their parent compounds because they have lower affinities for specific efflux pumps [43]. Tigecycline (11, Figure 3) is not significantly extruded by both Gram-negatives and Gram-positives [52] MFS efflux pumps, while telithromycin (12, Figure 3) shows increased activity toward bacterial strains characterized by elevated macrolide efflux [53].

EPIs have also been reported to inhibit the activity of some *M. tuberculosis* efflux pumps, both in vitro and in vivo [54].

In this context, in a recent study conducted by Pieroni et al. it was evaluated and demonstrated how compounds interfering with bacterial energy metabolism might be combined with the current therapy since their ability to affect drugs’ efflux. Indeed, in *M. tuberculosis*, one of the most relevant drug resistance mechanisms is associated with the ability of the pathogens to develop several efflux systems endowed with the ability to extrude different antibiotic classes.

This study analyzed the synthesis of several Thioridazine (TZ) derivatives (14–17, Figure 3), and their biological activity as efflux inhibitors together with the synergistic effect when administered with already known anti-tuberculosis drugs, both in vitro and on infected human monocyte-derived macrophages. The aim of this work was to modify TZ (13, Figure 3) in order to develop a compound with improved antibiotic adjuvant properties while decreasing the side effects, especially at the central nervous system (CNS) level.

Thirteen small molecules were rationally synthesized and their activities were compared with TZ. Such newly synthetized compounds resulted as less toxic toward human macrophages, while preserving high synergistic activity when administrated in combination with the first-line (isoniazid, rifampicin) drugs currently used for the treatment of tuberculosis [55].

## 3. Targeting β-Lactamases

The production of enzymes able to deactivate antibiotics is one of the resistance mechanisms used by bacteria to withstand the effect of antibiotic drugs [56].

β-lactam antibiotics, the first natural antibacterial compounds successfully developed, and subsequently modified, still represent a very important class of antibiotics thanks to their antibacterial activity and selectivity. However, their introduction to the market has been rapidly followed by the spreading of resistant strains on a global scale. In Gram-positive bacteria, hydrolysis of the first commercial penicillin by β-lactamases was the first reported resistance mechanism to β-lactams.

The mechanism of action through which these antibiotics work involves the inactivation of transpeptidases that are required for the last step of bacterial cell wall biosynthesis. Bacterial strains produce β-lactamases, which destroy the β-lactam ring, leading to a loss of efficacy of antibiotics containing this functional group.

The β-lactam ring is the key element responsible for antibiotic activity because of its electrophilicity, through which it irreversibly acylates the penicillin-binding proteins (PBPs). PBPs are responsible for the synthesis of peptidoglycan that is responsible for the structural integrity of the bacterial cell wall. To preserve the cell wall, the β-lactamases synthesized by bacteria are able to hydrolyze β-lactams-based antibiotics through the generation of an inactive open cycle, and the degree and the extent of hydrolysis depends on the type (and quantity) of beta-lactamases produced by the microorganisms. Gram-negative release β-lactamases in the periplasmatic space to prevent the antibiotic from reaching its target in the cytoplasmatic membrane [10], while Gram-positive bacteria release these enzymes in the extracellular space.

Nowadays, hundreds of β-lactamases have been discovered to be endowed with the same general mechanism of action. They differ from each other for the amino acid sequences and this has led to a different affinity for different substrates. In general, β-lactamases are classified in two different methods: One is Ambler classification and is based on a structural characterization, the other one is Bush and Jacoby classification and is based on a functional characterization [10,57].

The huge amount of β-lactams antibiotics used in therapy has promoted the synthesis of a particular class of β-lactamases, called extended-spectrum β-lactamases (ESBL), which are able to hydrolyze most β-lactam antibiotics, and these are characterized especially in *Enterobacteriaceae*, such as *E. coli*, *K. pneumoniae*, and *P. mirabilis* [58,59]. Carbapenemases represent the most versatile family of β-lactamases, with a broader spectrum if compared to other β-lactam-hydrolyzing enzymes. Many of these enzymes recognize almost all hydrolyzable β-lactams, and most are resistant to inhibition by all commercially viable β-lactamase inhibitors [60].

In this context, due to the growing increase in newly discovered β-lactamases, there is an urgent need of new and effective β-lactamases inhibitors as antibiotic adjuvants in antibiotic therapy [56].

Two strategies are pursued to overcome β-lactamase-mediated resistance to β-lactams: (i) The development of β-lactamase-stable antibiotics, for example cephalosporins and carbapenems, that are stable to hydrolysis by β-lactamases, and (ii) the development of selective β-lactamase inhibitors (BLIs) to be used in co-administration with a β-lactam antibiotic.

The selection of the inhibitor that can be combined with a specific β-lactam antibiotic is a complex step that takes into consideration different requirements: a) The ability of the inhibitors to protect the antibiotic against enzymatic hydrolysis, b) the dose of inhibitor required to ensure this protection, and c) the viability and stability of the combination.

**Figure 4:**
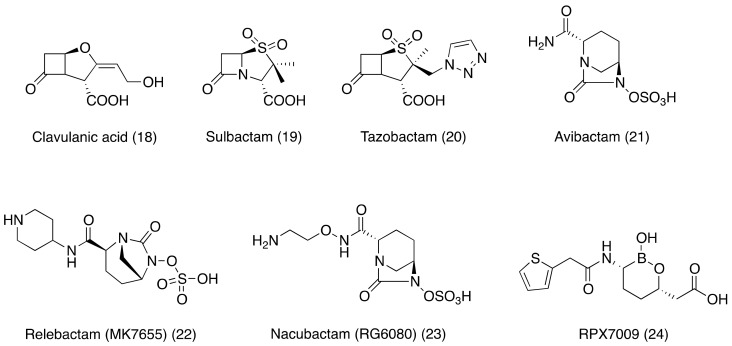
Chemical structures of β-lactamases inhibitors discussed in this review.

In this scenario, the discovery of clavulanic acid (18, Figure 4), a *Streptomyces clavuligerus* secondary metabolite, accounted for an important step in the field of antibacterial discovery. This β-lactam is capable to inactivate most of the β-lactamases, showing good antibacterial activity. This led to the development of the first β-lactam-β-lactamase inhibitor combination, Augmentin (amoxycillin/clavulanic acid) [61]. This combination of an antibiotic molecule with an antibiotic adjuvant received great commercial success and was followed by the introduction of other combinations.

Indeed, following the discovery of clavulanic acid, a medicinal chemistry campaign was started aiming at the synthesis of several penicillanic acid sulfones endowed with β-lactamase inhibitory activity. Among these, sulbactam and tazobactam (19 and 20, Figure 4) were successfully commercialized. Both had a similar spectrum of activity as clavulanic acid. Sulbactam is combined with ampicillin for global use and with cefoperazone to afford, in addition, a synergistic activity against anaerobic bacteria [62]. Tazobactam is combined with piperacillin and, recently, with cefoperazone and ceftolozane for nosocomial infections, including those caused by MDR *P. aeruginosa* [63]. In general, these compounds do not show an antibacterial activity if administered alone, but there are several exceptions. It has been demonstrated that clavulanic acid alone has a MIC as low as 1 µg/mL against *N. gonorrhoeae* [64]. Sulbactam has a low activity against wild-type *Acinetobacter spp.* and *Burkholderia cepacian*, with MIC values <8 and 10 µg/mL, respectively, but does not show activity against strains with multiple resistance mechanisms [65].

The first discoveries of β-lactamase inhibitors were followed by a gap of two decades. Then, a class of non-β-lactam β-lactamase inhibitors, based on the diazabicyclooctane (DBO) scaffold, arose. Avibactam is the first inhibitor of this class. It has a larger spectrum of activity compared to clavulanic acid. Avibactam (21, Figure 4) has been approved for its use in therapy in combination with ceftazidime, and the development of other combinations is ongoing, such as ceftaroline-avibactam or aztreonam-avibactam combinations [66,67,68]. Other DBOs under development include Nacubactam (RG6080, 22 Figure 4) and relebactam (MK7655, 23, Figure 4), in combination with imipenem. The spectrum of relebactam is similar to the spectrum of activity of avibactam. RG6080 (formerly OP0565) is a DBO endowed with an inhibitory spectrum similar to the other DBOs but it exhibits some intrinsic antibacterial activity against enteric bacteria [69].

Another new class of synthetic non β-lactam β-lactamase inhibitors are made up of boronic acids [70]. Among these, RPX7009 (24, Figure 4) is developed in combination with meropenem to target pathogens that synthesize carbapenemases [71].

Resistance to β-lactams continues to spread, especially in Gram-negative organisms [72,73]. Nowadays, the most pursued challenge in this field is to deal with resistance developing new β-lactamase inhibitors that will provide protection for the most used antibiotics in clinical therapies, at least for the present time.

## 4. Targeting Outer Membrane

Generally, the antibiotics used in therapy exert their antibacterial action, hitting the respective targets inside the cell. This requires the antibiotics to cross the bacterial membrane(s), to be able to reach their target(s). To withstand the permeation of antibiotics, Gram-negative are protected by the presence of a further level of defense, which is the outer membrane [74].

Indeed, the Gram-negative’s outer membrane, which is mainly composed of polyanionic lipopolysaccharides and porins, limits the entrance of xenobiotic such as antibiotics. As a consequence, some antibacterials show reduced efficacy in treating Gram-negative infections because of the complex architecture of their wall.

As a matter of fact, the complex architecture and composition of the membrane wall heavily affects the bacterium vulnerability toward the antibiotic. It is not surprising, therefore, that most of the occurring resistant strains usually adopt protein mutations, at the outer membrane level, in order to overcome the action of some antibacterial drugs.

For penetration across the bacterial wall, antibiotics use two different strategies, depending on the chemical nature of the small-molecule:

- Hydrophobic compound (such as macrolides and rifampicin) cross the lipid bilayer through passive transport mechanisms.
- Hydrophilic molecules (such as β-lactams, fluoroquinolones, and phenicol antibiotics) diffuse through active transport mechanisms, taking advantage of their ability to interact with peculiar porins [74,75].

In this context, the bacterial outer membrane represents a potential target able to deal with bacterial resistance, and a better knowledge of its structure will surely increase our ability to develop new classes of antibiotics, endowed with a peculiar mechanism of actions [76].

**Figure 5:**
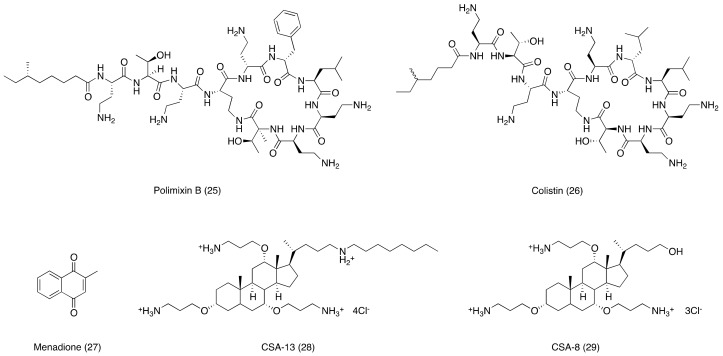
Chemical structures of membrane permeabilizers discussed in this review.

The use of antibiotic adjuvants to increase the membrane permeability propensity (e.g., permeabilizer) proved to be a good strategy to improve the entrance of antibiotics. In general, permeabilizers are cationic and amphiphilic molecules or chelators that, by interacting with polyanionic lipopolysaccharides or by capturing outer layer cations, destabilize the membrane wall. Consequently, the outer membrane becomes more prone to being crossed by xenobiotic. Examples of outer membrane permeabilizers are polymyxins, such as polymyxin B (25, Figure 5), colistin (26, Figure 5), aminoglycosides, cationic peptides, cationic cholic acid derivatives, or polyamines [77,78].

Alternative strategies to design or to identify new small molecules that could facilitate the diffusion of antibiotics, increasing their intracellular concentration, are of great demand [79]. In this scenario, several chemosensitizers able to disrupt membrane protein activities (e.g., porins and membrane channels) have been proposed (e.g., detergents, surfactants, antimicrobial peptides, etc.) [80,81]. Such classes of adjuvants have been administrated in combination with classical antibiotics to fight with resistant strains [82,83].

Recently, it has been reported that a glycine basic peptide (GBP) exerts its antibacterial activity on the membrane of *E. coli* with a concentration-dependent mechanism of action. This study demonstrated that the use of GBP leads to a cell with fragmentation at a high concentration. This cationic peptide works by disrupting the membrane barrier and the *E. coli* ion-channel. The result is a loss of ions Ca^2+^, K^+^, and Mg^2+^. The use of GBP had improved the sensitivity of *E. coli* to erythromycin and rifampicin. Indeed, the aforementioned drugs resulted as unable to penetrate the intact outer membrane of Gram-negative bacteria. On the contrary, when the membrane is damaged (e.g., by the use of GBP) they are capable of getting inside the bacterial cell, engaging the target(s) for which they were originally designed. These observations support the notion that GBP works as permeabilizers, increasing the membrane permeability, and in turn, facilitating the penetration of antibiotics [84].

Another study was conducted regarding the evaluation of menadione (27, Figure 5) and its effect on the membrane permeability of MDR strains of *S. aureus, P. aeruginosa*, and *E. coli*. At the intestinal level, menadione is converted to vitamin K_2_, being characterized by antibacterial activity only against the *P. aeruginosa* strain. Surprisingly, menadione, in combination with antibiotics from the aminoglycoside family, provided a reduction of the inhibitory concentration of these antibiotics and suggested a synergistic effect in combination therapy [85].

Another class of interesting molecules endowed with antibiotic adjuvant potential is represented by endogenous antimicrobial peptides (AMPs), which are factors secreted by host cells and organs (e.g., neutrophils, exocrine glands, etc.) representing the so-called defensive line in innate immunity against pathogens. The mechanism of action of AMPs is based on their ability to destabilize the outer cell membrane of prokaryotes by the formation of an amphipathic α-helix or short β-sheet structures [86], which act through fast and specific membrane dysfunction [87]. Nevertheless, their therapeutic use remains uncertain due to the high costs of their production, metabolic liability, and the consequent development of mechanisms of resistance by the bacteria. Indeed, the proteases secreted by bacteria have been shown to neutralize AMP’s activity [88,89].

In order to overcome the aforementioned issues linked to the use of AMPs, a new class of adjuvant, namely “Caragenins”, was developed. Caragenins are cationic steroidal antibiotics (CSA), in which the alkoxy groups of the sterol core structure is substituted by an aminoalkyl function. This structural modification makes “Caragenins” resistant to the action of proteases since they do not present peptidic bonds in their structure and they can be produced in large amounts. Moreover, CSA are able to stably incorporate into membranes, having the ability to form complexes with phospholipids [90,91]. On the contrary, CAS being positively charged assure their electrostatic attraction to the negatively charged membranes (bacteria, viruses, fungi, and protozoa), leading to cell death through disruption of the membrane [92]. Caragenins CSA-13 (28, Figure 5) and CSA-8 (29, Figure 5) were synthesized to mimic the cationic structural physico-chemical properties of AMPs, sharing a similar mechanism of action with them, based on (i) induction of a rapid depolarization of the bacterial membrane and (ii) increased permeabilization of the outer membranes of Gram-negative bacteria. In such a way, CSA-13 (28, Figure 5) and CSA-8 make the microorganisms more susceptible to hydrophobic antibiotics Indeed, the MIC of erythromycin used alone against a resistant strain of *K. pneumoniae* is 70 µg/mL, while the use of erythromycin in combination with compound CSA-8 leads to the reduction of its MIC to 1 μg/mL. It was also analyzed the anti-bactericidal activity of CSA-13 on 60 carbapenem-resistant strains. In this study, the authors demonstrated that by combining CSA-13 with antibiotics, synergy was achieved with colistin (55%) and tobramycin (35%), while no antagonism was observed [88,89]. It is worth mentioning that the development of this class of antibiotic adjuvants requires extensive research to improve the absorption, distribution, metabolism, and extrection (ADME) profile of such molecules, allowing them to enter clinical trials and in turn, to reach the market.

## 5. Targeting Antivirulence Factors

On the route to the identification of novel antibiotics active against drug-resistant strains, the motivation to consider alternative cellular pathways as a source of targets for the development of new antibacterial adjuvant classes represent an interesting alternative to classical approaches.

The resistance to antibiotics often causes latent and persistent infections that are very challenging to deal with. During persistence inside the host, pathogens face extremely hostile environments, thus requiring an extensive re-programming of the bacterial metabolism functions, in order to survive such adverse conditions. Therefore, to target key metabolic functions that are relevant for the pathogens to survive in such conditions, could result in better antibiotic treatments and increase susceptibility to traditional antibiotics.

In this context, the main factor for the degeneration of a patient’s health during a bacterial infection is bacterial virulence. Over the last decade, a new approach has emerged, designed to hit bacterial virulence, or pathogenicity [21,93,94,95]. In contrast to traditional antimicrobial drugs, which act by killing bacteria or blocking bacterial growth, antivirulence drugs hit specific targets called virulence factors that are expressed in bacteria only during infection. They are non-essential for the basal bacterial cell-cycle, but they are essential for pathogenesis and their pharmacological inactivation results in bacteria not being able to cause pathological infections in the host. In this context, the host immune system can promptly and easily work more successfully against less virulent [96] pathogens. In addition, antivirulence inhibitors do not target essential factors for the pathogen’s life cycle, it is assumed that in this context, the selection pressure for resistant mutants will be less relevant [97]. Among others, examples of pathways enriched with non-essential targets include the sulfur assimilation pathway, quorum sensing, and biofilms [98].

### 5.1. Targeting Cysteine Biosynthesis

The rationale behind the exploitation of amino acid biosynthesis as a target for antimicrobial adjuvant development is the observation that some pathogens spend part of their life cycle in extremely harsh conditions, such as macrophages or the gastric mucosa, where survival and proliferation require powerful adaptation mechanisms involving metabolic pathways [99,100]. In such conditions, interfering with pathogen adaptation strategies may lead to an increase in the susceptibility to antibiotics.

Among the possible new drug targets are the enzymes involved in the cysteine biosynthesis.

It has been observed that the importance of cysteine biosynthetic enzymes varies within the life cycle of pathogens: their activity can be dispensable during growth in vitro or acute infections but becomes indispensable during the persistence phase [101,102]. Molecules developed to target biosynthetic pathways like cysteine biosynthesis may have the potential advantage of fighting persistence inside the host more effectively than traditional antibiotics do, contributing to the prevention of resistance development that takes place during clinical latency [103,104].

Many studies on the response of microorganisms to environmental stress (such as nutrient starvation, hypoxia, oxidative stress) point to an upregulation of many genes of the cysteine regulon.

Thus, a role for cysteine biosynthesis in the development of antibiotic resistance has recently been pointed out [105,106]. Investigations on deletion mutants of the cysteine biosynthetic pathway in *S. typhimurium* led to the conclusion that an unpaired oxidative stress response, due to inhibition of cysteine biosynthesis, causes a decrease in antibiotic resistance, in both vegetative and swarm cell populations. Antibiotic-induced oxidative stress, which is widely recognized as a general mechanism of action of many antibacterials [107], could explain the reduced resistance rate observed in bacteria with impaired cysteine biosynthesis. These findings suggest that inhibitors of cysteine biosynthesis could thus enhance the efficacy of antibiotic treatment and decrease the spreading of resistance [101].

Most bacteria and plants carry out cysteine biosynthesis by the reductive sulfate assimilation pathway (RSAP), a multistep reduction of sulfate that culminates in the incorporation of bisulfide into cysteine using an activated form of serine [108].

RSAP begins with the transportation of sulfate inside the cell, followed by its reduction to bisulfide. This process is highly energy-consuming and is tuned to cellular needs. Bacteria find sulfur in the form of sulfate in the environment and actively transport it through plasma membrane.

After the reduction of sulfate to bisulfide, a toxic compound whose concentration inside the cell is kept between 20 and 160 µM, the latter is incorporated into cysteine by a member of a large enzyme family, known as cysteine synthase complex (CSC). The CSC is composed by the enzyme serine acetyl transferase (SAT) and *O*-acetylserine sulfydrylase (OASS), which catalyzes the last step of cysteine biosynthesis [109].

SAT is catalytically active in the complex and produces *O*-acetyl-l-serine (OAS), the activated form of serine, a cysteine precursor where the β-hydroxyl group of the amino acid is acetylated or phosphorylated to generate a better living group in the β-elimination reaction.

OAS is unstable and spontaneously converts to *N*-acetylserine (NAS), the natural inducer of the cysteine regulon signaling. SAT catalyzes the reaction by which an acetyl group from acetyl-CoA is transferred to the hydroxyl one of l-serine to form OAS and CoA. The C-terminus tail of SAT is fundamental for function and regulation, being responsible for an intrasteric inhibition in the presence of cysteine by binding to OASS for the formation of CSC [110].

**Figure 6:**
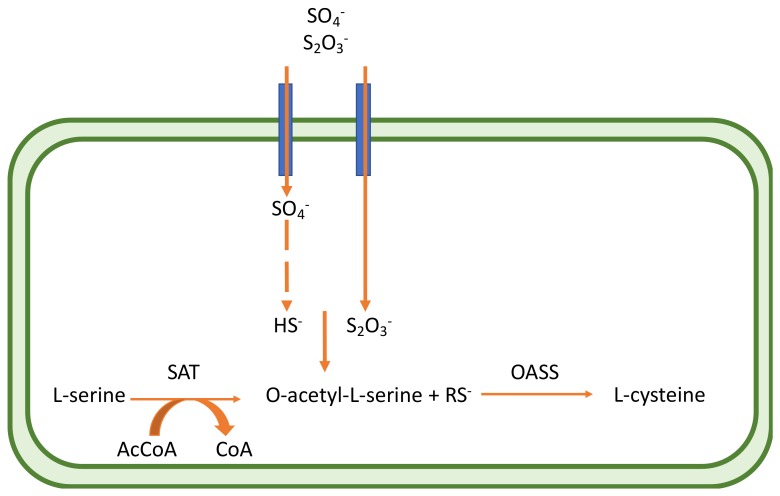
Schematic representation of the Cysteine-synthase-complex (CSC) within bacterial cell; RSAP begins with the transportation of sulfate inside the cell, followed by its reduction to bisulfide. This process is highly energy-consuming and is tuned to cellular needs. Bacteria find sulfur in the form of sulfate in the environment and actively transport it through plasma membrane. After the reduction of sulfate to bisulfide, the latter is incorporated into cysteine by a member of a large enzyme family, known as cysteine synthase complex (CSC). The CSC is composed by the enzyme serine acetyl transferase (SAT) and O-acetylserine sulfydrylase (OASS), which catalyzes the last step of cysteine biosynthesis.

Depending on the organism and growth conditions, the last step of cysteine biosynthesis is catalyzed by different sulfydrylases, that share high homology but also show some functional and structural differences. The first identified OASS isoform (OASS-A) was firstly isolated and characterized from *Salmonella typhimurium*. Later it was observed how many pathogens possess two isoforms of the enzyme: *O*-acetylserine sulfydrylase (OASS-A encoded by *cysK*) and *O*-phosphoserine sulfydrylase (OASS-B, encoded by *cysM*), which differ for peculiar functional and structural properties. In *S. typhimurium*, both OASS-A and B isoforms use *O*-acetylserine (OAS) as a substrate and S^2−^ as a primary sulfur source. The OASS isoenzymes are differently expressed depending on the environment conditions (OASS-A in aerobic conditions, while OASS_B in an anaerobic environment) [109]. They are both PLP-dependent enzymes, belonging to the fold type II sub-family of the PLP-enzyme superfamily. They also share the same Bi-Bi ping pong-type reaction mechanism, where the first half-reaction is the generation of the α-aminoacrylate intermediate Schiff base through the β-elimination of the β-substituted l-serine external aldimine. In the second half-reaction, the sulfur source attacks the α-aminoacrylate in order to give l-Cys [111]. Since its discovery, OASS was thought to be involved in plural functions, such as swarming motility in *Proteus mirabilis* [112], transcriptional regulation in some bacteria and nematodes [113], toxin activation in *E. coli* [114], and antibiotic resistance in *S. typhimurium* [105,106] (Figure 6).

According to these facts, and taking into account that the concomitant inhibition of both enzymes is required to block the biosynthesis of l-Cys [110], the identification of small-molecules able to inhibit both OASS isoforms is valuable to investigate and support the physiological role of these enzymes in bacteria’s life cycle.

**Figure 7:**
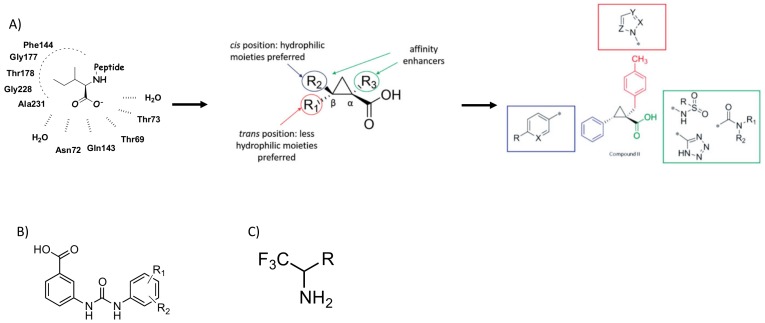
*O*-Acetylserine sulfhydrilase (OASS) inhibitors discussed in this review. (**A**) schematic representation of the rational design of the first sulfhydrylase inhibitors based on the structural features of the OASS–SAT interaction; (**B**) chemical scaffold of potent inhibitors of CysM, enzyme in cysteine biosynthesis during mycobacterium dormancy; (**C**) chemical scaffold of fluoroalanine derivatives endowed with the ability to irreversibly inhibit both OASS-A and B isozymes.

In 2016, Pieroni et al. [115,116], starting from the evidence that SAT competitively inhibits OASS-A, developed a rational design of the first sulfydrylase inhibitors based on the structural features of the OASS–SAT interaction. Taking into account the data from their previous studies [117], and combining computational [118,119] and spectroscopic approaches, such as saturation transfer difference (STD) and nuclear magnetic resonance (NMR), they rationally designed and synthesized a series of 2-phenylcyclopropane carboxylic acid derivatives tested against both isoforms of *St*-OASS [120]. Indeed, they demonstrated that the compounds binding to the enzyme active sites efficiently inhibit both OASS-A and B isoforms by competing with SAT. These findings provide a proof of principle, supporting the notion that it is possible to develop small molecules able to inhibit both OASS enzymes and paving the way to develop pharmacological tools enabling the investigation of such enzymes as pharmaceutical targets to deal with bacterial virulence and drug resistance (Figure 7A).

In this context, in 2016, Brunner et al. [121] reported the identification and characterization of potent inhibitors of CysM, a critical enzyme in cysteine biosynthesis during mycobacterium dormancy. In their work, Brunner at al. screened 17,312 compounds in order to identify CysM inhibitors, disclosing urea derivatives able to bind to the CysM active site in the µM range. The hit identified by such an approach bind CysM with an affinity of 300 nM, also resulting as selective against the homologous CysK1 and CysK2. Finally, two inhibitors turned out to be active in a nutrient-starvation model of dormancy of *M. tuberculosis*, with little or no cytotoxicity toward mammalian cells (Figure 7B).

In 2018, Franko et al. [122] investigated fluoroalanine derivatives for their ability to irreversibly inhibit both OASS-A and B isozymes. The starting hypothesis was based on the observation that mono-, di-, and tri-alogenenated alanines have already been reported as mechanism-based inhibitors of PLP-dependent enzymes, such as γ-cystathionase [123,124], alanine racemase [125], tryptophan synthase and tryptophanase [123,126], 8-amino 7-oxonononatote synthase [127], ornithine decarboxylase [128], aspartate aminotransferase [129], and kynurenine transaminase [130]. Following spectra changes, the authors demonstrated that monofluoroalanine reacts with the OASS-A and OASS-B, forming both stable and metastable α-aminoacrylate Schiff’s base. As previously demonstrated for other β-halogenalanine derivatives, these findings support the notion that monofluoroalanine works as a substrate analogue. For both OASS isoforms, trifluoroalanine derivative induced spectral changes and a biphasic behavior of the time course of enzyme inactivation associated with an irreversible inhibition mechanism.

The authors concluded that monofluoralanine is a weak substrate analogue for both OASS-A and B, on the contrary, trifluoroalanine acts as an irreversible inhibitor, although it is poorly efficient (Figure 7C).

### 5.2. Targeting Quorum Sensing

In many bacterial pathogens, the population growth is under the control of quorum sensing (QS), which is a cell–cell communication mechanism controlling phenotype manifestations, such as virulence [131]. This system is employed by bacteria to communicate with each other in a given population [132]. It consists of a constant secretion of signal molecules (called autoinducers) by each individual bacterium, and the QS-controlled process is activated when a defined concentration of this molecular messenger reaches a threshold. Several virulence aspects are influenced by QS, and for this reason, the identification of small molecules able to interfere with such mechanisms of cell–cell is currently a field of great interest.

QS has been identified in a broad range of both Gram-negative and Gram-positive bacteria. It has a great importance in many pathogenic species and has been shown to also play a role in biofilm formation [133]. In Gram-positive bacteria, the signal molecules consisted in peptides, while Gram-negative bacteria use N-acylhomoserine lactones (AHLs) that differ from each other with subtle differences in chemical structure, as well as other autoinducers. Due to these similarities, through this mechanism, bacteria can communicate between species, and this is useful for the bacterial co-infections. Often, in one species, multiple communication systems exist, that are interconnected and influence each other [134]. In the literature, several reviews, describing various quorum sensing systems, are reported [135,136].

Azithromycin, which does not have a bactericidal effect on pseudomonas aeruginosa and interferes with its QS pathways, was studied in clinical trials [137]. In their study, Kohler et al. showed that azithromycin reduces the presence of QS signals in vitro and in vivo. Thus, it may help in acute infections to reduce virulence.

**Figure 8:**
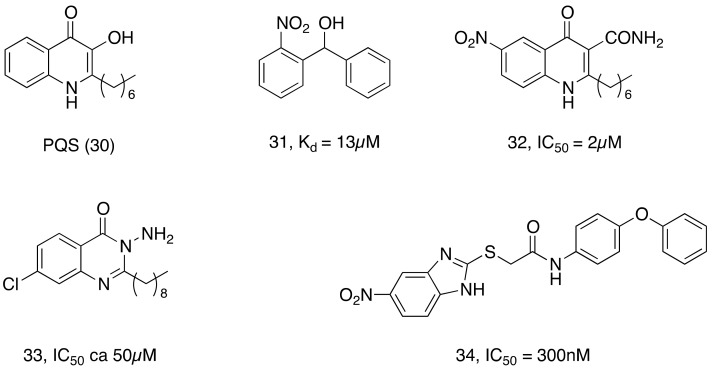
Chemical structures of Quorum sensing (QS) inhibitors discussed in this review.

Several approaches for developing quorum sensing inhibitors start by mimicking the chemical structure of quorum sensing signal molecules. In this context, the *pseudomonas* quinolone signal (PQS) system of *P. aeruginosa* can be considered a potential target. The biosynthesis of the PQS signal 30 requires the action of a set of enzymes PqsABCDEH and its autocatalytic receptor PqsR (MvfR). It has been demonstrated that compound 31 acts as PqsD transition state analogues and reduces bacterial biofilm formation. Later, it was shown that numerous compounds, such as 32, 33, 34, and 35 (Figure 8), inhibited the QS system and biofilm formation in infection models [138,139].

### 5.3. Targeting Biofilms

In almost 65% of infections, bacteria grow as biofilms, which are communities of microorganisms growing on a surface. In the growth phase, under infection conditions, bacteria became 10- to 1000-fold more resistant to antibiotics.

Current biofilm-targeting approaches can be divided into two groups: the first is a physical-mechanical approach that is aimed at disrupting and removing the biofilms and the second approach is consisted in the use of antibiotics or antimicrobials on a matrix for biofilm prevention [140].

As a result, peptide 1018 can be used as a new class of antibiotic adjuvants that not only have broad spectrum activity [141], but can also be synergized with commonly used antibiotics, such as tobramycin, ceftazidime, imipenem, and ciprofloxacin [142].

However, the biofilm-targeting therapeutics field is quickly growing, and nowadays, it includes different strategies aimed at interfering with such bacterial cell to cell communication mechanisms. The topic is largely and deeply discussed in other interesting and recent reviews that are listed subsequently. These reviews focus on the challenges in the development of anti-biofilm strategies and on the identification of the role of dormant persister cells in antibiotic tolerance. They also focus on technologies with an efficacy profile in preclinical trials, or in robust animal or human cell infection models [143,144,145,146,147].

Future research will be focused on reaching a good efficacy and specificity together with low toxicity to develop low-cost and practical formulations for clinical use.

## 6. Conclusions

For a number of human and animal pathogens, AMR is considered as one of the most serious current threats for global health. Infections caused by resistant microorganisms are difficult to treat, often requiring costly and sometimes toxic alternatives to common therapeutic strategies. Problems related to AMR are expected to become more dramatic unless worldwide efforts are undertaken in order to preserve and/or increase the currently available antimicrobial drugs arsenal.

Among many strategies explored to date, one solution to the emerging AMR phenomenon is represented by combination therapies of existing antibiotics and smart antibiotic adjuvants. In such a case, via a multi-targeted approach, an old ineffective antibiotic agent becomes efficacious again against the resistant strain of interest.

A well-known example is represented by the class of β-lactamase inhibitors, that resulted to be efficacious in expanding the spectrum of the existing β-lactam antibiotics.

Another emerging class of drug-targets that recently received significant attention is the efflux pump family. Indeed, efflux pumps are ubiquitously expressed in different pathogens and they are largely involved in resistance to many different antibiotic classes in both Gram-positive and Gram-negative bacteria, making them an attractive class of drug-targets. Several efflux pump inhibitors have been disclosed so far; nevertheless, additional work needs to be done in order to improve the safety profile of such a class of antibiotic adjuvants.

The new classes of amphipathic molecules provide the opportunity to target bacterial membranes through both hydrophilic substituents (polyamines), which are able to interact with negative charges present in the surface of the membrane, and hydrophobic substituents, which interact with and disrupt the lipid chains of the bacterial membrane. Through the antibiotic adjuvant strategy, a polypharmacy approach on bacteria can be performed, not only by providing the entrance of the antibiotics inside the cell but also by de-energizing the efflux pumps used by the bacteria to escape antibiotic action.

The identification of molecules that inhibit sulfur metabolism, as they are expected to strongly decrease bacterial fitness at infection loci, is a promising strategy to increase the efficacy of existing antibiotics acting as potential antibiotic adjuvants, thus providing a new life to old molecules.

It is important to note that the list of antibiotic adjuvant approaches described in this review is not complete, indeed, (i) among the known pathways there are many undrugged targets, which still require the development of a probe in order to be validated and in turn, represent new pharmaceutical targets. (ii) In the advent of the “omic” era, there are undiscovered pathways that could be targeted.

In conclusion, in the near future, AMR will continue to be one of the main threats for global health, which will require continuous and significant efforts at different social levels. Therefore, the identification of new strategies to limit or to overcome the occurrence of resistance strains will be a long journey, where antibiotic adjuvants counteracting antibiotic resistances will cover a significant area of the AMR fields.

## Figures and Tables

**Table 1:** Antibiotic adjuvants classes and their inhibitors reported in this review.

Antibiotiv Adjuvant Class		Compound Name	Bacterium
*Efflux Pump Inhibitors*		Phenotiazines, Phenylalanine-arginine-β-naphtylamide (PaβN), Arylpiperazine, Quinolines, Thioridazine (TZ) derivatives	Gram-positive Gram-negative
*β*-Lactamase inhibitors		Clavulanic acid, Sulbactam, Tazobactam, Diazabicyclooctane (DBO) Boronic acids	Gram-positive Gram-negative
*Membrane Permeabilizers*		Polimixyn B Colistin Aminoglycosides Polycationic/cationic antimicrobial peptides Glycine basic peptide (GBP) Caragenins Menadione	Gram-positive Gram-negative
*Antivirulence Compounds*	Reductive Sulfur Assimilation pathway	OASS-inhibitors, SAT-inhibitors, Cys-inhibitors	Gram-positive Gram-negative
	Quorum Sensing	PqsD transition state analogues	
	Biofilm	physical-mechanical approach antibiotics or antimicrobials on a matrix peptide 1018	

## References

[1] Davies J., Davies D. (2010). Origins and Evolution of Antibiotic Resistance. Microbiol. Mol. Biol. Rev..

[2] Aminov R.I. (2010). A Brief History of the Antibiotic Era: Lessons Learned and Challenges for the Future. Front. Microbiol..

[3] Singh S.B. (2014). Confronting the challenges of discovery of novel antibacterial agents. Bioorg. Med. Chem. Lett..

[4] Reder-Christ K., Bendas G. (2011). Biosensor Applications in the Field of Antibiotic Research—A Review of Recent Developments. Sensors.

[5] Projan S.J., Shlaes D.M. (2004). Antibacterial drug discovery: Is it all downhill from here?. Clin. Microbiol. Infect..

[6] Spellberg B., Gilbert D.N. (2014). The Future of Antibiotics and Resistance: A Tribute to a Career of Leadership by John Bartlett. Clin. Infect. Dis..

[7] Fair R.J., Tor Y. (2014). Antibiotics and Bacterial Resistance in the 21st Century. Perspect. Med. Chem..

[8] Aslam B., Wang W., Arshad M.I., Khurshid M., Muzammil S., Rasool M.H., Nisar M.A., Alvi R.F., Aslam M.A., Qamar M.U. (2018). Antibiotic resistance: A rundown of a global crisis. Infect. Drug Resist..

[9] Admassie M. (2018). Current Review on Molecular and Phenotypic Mechanism of Bacterial Resistance to Antibiotic. Sci. J. Clin. Med..

[10] Walsh C. (2000). Molecular mechanisms that confer antibacterial drug resistance. Nature.

[11] González-Bello C. (2017). Antibiotic adjuvants—A strategy to unlock bacterial resistance to antibiotics. Bioorg. Med. Chem. Lett..

[12] Cassini A., Högberg L.D., Plachouras D., Quattrocchi A., Hoxha A., Simonsen G.S., Colomb-Cotinat M., Kretzschmar M.E., Devleesschauwer B., Cecchini M. (2019). Attributable deaths and disability-adjusted life-years caused by infections with antibiotic-resistant bacteria in the EU and the European Economic Area in 2015: A population-level modelling analysis. Lancet Infect. Dis..

[13] Blair J.M.A., Webber M.A., Baylay A.J., Ogbolu D.O., Piddock L.J.V. (2015). Molecular mechanisms of antibiotic resistance. Nat. Rev. Microbiol..

[14] Bush K., Courvalin P., Dantas G., Davies J., Eisenstein B., Huovinen P., Jacoby G.A., Kishony R., Kreiswirth B.N., Kutter E. (2011). Tackling antibiotic resistance. Nat. Rev. Microbiol..

[15] Goff D.A., Kullar R., Goldstein E.J.C., Gilchrist M., Nathwani D., Cheng A.C., Cairns K.A., Escandón-Vargas K., Villegas M.V., Brink A. (2017). A global call from five countries to collaborate in antibiotic stewardship: United we succeed, divided we might fail. Lancet Infect. Dis..

[16] Tommasi R., Brown D.G., Walkup G.K., Manchester J.I., Miller A.A. (2015). ESKAPEing the labyrinth of antibacterial discovery. Nat. Rev. Drug Discov..

[17] Ventola C.L. (2015). The Antibiotic Resistance Crisis. Pharm. Ther..

[18] Laxminarayan R., Duse A., Wattal C., Zaidi A.K.M., Wertheim H.F.L., Sumpradit N., Vlieghe E., Hara G.L., Gould I.M., Goossens H. (2013). Antibiotic resistance—The need for global solutions. Lancet Infect. Dis..

[19] Wright G.D. (2016). Antibiotic Adjuvants: Rescuing Antibiotics from Resistance. Trends Microbiol..

[20] Hartzell J.D., Neff R., Ake J., Howard R., Olson S., Paolino K., Vishnepolsky M., Weintrob A., Wortmann G. (2009). Nephrotoxicity Associated with Intravenous Colistin (Colistimethate Sodium) Treatment at a Tertiary Care Medical Center. Clin. Infect. Dis..

[21] Clatworthy A.E., Pierson E., Hung D.T. (2007). Targeting virulence: A new paradigm for antimicrobial therapy. Nat. Chem. Biol..

[22] Garland M., Loscher S., Bogyo M. (2017). Chemical Strategies to Target Bacterial Virulence. Chem. Rev..

[23] Nikaido H., Zgurskaya H.I. (1999). Antibiotic efflux mechanisms. Curr. Opin. Infect. Dis..

[24] Webber M.A. (2003). The importance of efflux pumps in bacterial antibiotic resistance. J. Antimicrob. Chemother..

[25] Ramos J.L., Duque E., Gallegos M.-T., Godoy P., Ramos-González M.I., Rojas A., Terán W., Segura A. (2002). Mechanisms of Solvent Tolerance in Gram-Negative Bacteria. Annu. Rev. Microbiol..

[26] Zgurskaya H.I., Nikaido H. (2000). Multi-drug resistance mechanisms: Drug efflux across two membranes. Mol. Microbiol..

[27] Nies D.H. (2003). Efflux-mediated heavy metal resistance in prokaryotes. FEMS Microbiol. Rev..

[28] Pagès J.-M., Masi M., Barbe J. (2005). Inhibitors of efflux pumps in Gram-negative bacteria. Trends Mol. Med..

[29] Saier M.H., Paulsen I.T. (2001). Phylogeny of multi-drug transporters. Semin. Cell Dev. Biol..

[30] Morita Y., Tomida J., Kawamura Y. (2014). Responses of Pseudomonas aeruginosa to antimicrobials. Front. Microbiol..

[31] Zhanel G.G., Hoban D.J., Schurek K., Karlowsky J.A. (2004). Role of efflux mechanisms on fluoroquinolone resistance in Streptococcus pneumoniae and Pseudomonas aeruginosa. Int. J. Antimicrob. Agents.

[32] Davin-Regli A., Bolla J.-M., James C.E., Lavigne J.-P., Chevalier J., Garnotel E., Molitor A., Pagès J.-M. (2008). Membrane permeability and regulation of drug “influx and efflux” in enterobacterial pathogens. Curr. Drug Targets.

[33] Lomovskaya O., Lee A., Hoshino K., Ishida H., Mistry A., Warren M.S., Boyer E., Chamberland S., Lee V.J. (1999). Use of a genetic approach to evaluate the consequences of inhibition of efflux pumps in Pseudomonas aeruginosa. Antimicrob. Agents Chemother..

[34] McMurry L., Petrucci R.E., Levy S.B. (1980). Active efflux of tetracycline encoded by four genetically different tetracycline resistance determinants in *Escherichia coli*. Proc. Natl. Acad. Sci. USA.

[35] Kumar S., Mukherjee M.M., Varela M.F. (2013). Modulation of Bacterial Multi-drug Resistance Efflux Pumps of the Major Facilitator Superfamily. Int. J. Bacteriol..

[36] Bolla J.-M., Alibert-Franco S., Handzlik J., Chevalier J., Mahamoud A., Boyer G., Kieć-Kononowicz K., Pagès J.-M. (2011). Strategies for bypassing the membrane barrier in multi-drug-resistant Gram-negative bacteria. FEBS Lett..

[37] Adams K.N., Takaki K., Connolly L.E., Wiedenhoft H., Winglee K., Humbert O., Edelstein P.H., Cosma C.L., Ramakrishnan L. (2011). Drug Tolerance in Replicating Mycobacteria Mediated by a Macrophage-Induced Efflux Mechanism. Cell.

[38] Adams K.N., Szumowski J.D., Ramakrishnan L. (2014). Verapamil, and Its Metabolite Norverapamil, Inhibit Macrophage-induced, Bacterial Efflux Pump-mediated Tolerance to Multiple Anti-tubercular Drugs. J. Infect. Dis..

[39] Kamicker B.J., Sweeney M.T., Kaczmarek F., Dib-Haj F., Shang W., Crimin K., Duignan J., Gootz T.D., Champney W.S. (2008). Bacterial Efflux Pump Inhibitors. New Antibiotic Targets.

[40] Zechini B., Versace I. (2009). Inhibitors of multi-drug-resistant efflux systems in bacteria. Recent Patents Anti-Infect. Drug Disc..

[41] Neyfakh A.A., Borsch C.M., Kaatz G.W. (1993). Fluoroquinolone resistance protein NorA of *Staphylococcus aureus* is a multi-drug efflux transporter. Antimicrob. Agents Chemother..

[42] P Tegos G., Haynes M., Jacob Strouse J., Md T Khan M., G Bologa C., I Oprea T., A Sklar L. (2011). Microbial Efflux Pump Inhibition: Tactics and Strategies. Curr. Pharm. Des..

[43] Lynch A.S. (2006). Efflux systems in bacterial pathogens: An opportunity for therapeutic intervention? An industry view. Biochem. Pharmacol..

[44] Tohidpour A., Najar Peerayeh S., Mehrabadi J.F., Rezaei Yazdi H. (2009). Determination of the Efflux Pump-Mediated Resistance Prevalence in *Pseudomonas aeruginosa*, Using an Efflux Pump Inhibitor. Curr. Microbiol..

[45] Kanagaratnam R., Sheikh R., Alharbi F., Kwon D.H. (2017). An efflux pump (MexAB-OprM) of *Pseudomonas aeruginosa* is associated with antibacterial activity of Epigallocatechin-3-gallate (EGCG). Phytomedicine.

[46] Chan Y.Y., Ong Y.M., Chua K.L. (2007). Synergistic interaction between phenothiazines and antimicrobial agents against *Burkholderia pseudomallei*. Antimicrob. Agents Chemother..

[47] Bailey A.M., Paulsen I.T., Piddock L.J.V. (2008). RamA Confers Multi-drug Resistance in Salmonella enterica via Increased Expression of acrB, Which Is Inhibited by Chlorpromazine. Antimicrob. Agents Chemother..

[48] Mahamoud A., Chevalier J., Davin-Regli A., Barbe J., Pagès J.-M. (2006). Quinoline derivatives as promising inhibitors of antibiotic efflux pump in multi-drug-resistant *Enterobacter aerogenes* isolates. Curr. Drug Targets.

[49] Pradel E., Pages J.-M. (2002). The AcrAB-TolC Efflux Pump Contributes to Multi-drug Resistance in the Nosocomial Pathogen *Enterobacter aerogenes*. Antimicrob. Agents Chemother..

[50] Bohnert J.A., Kern W.V. (2005). Selected Arylpiperazines Are Capable of Reversing Multi-drug Resistance in *Escherichia coli* Overexpressing RND Efflux Pumps. Antimicrob. Agents Chemother..

[51] Schumacher A., Steinke P., Bohnert J.A., Akova M., Jonas D., Kern W.V. (2006). Effect of 1-(1-naphthylmethyl)-piperazine, a novel putative efflux pump inhibitor, on antimicrobial drug susceptibility in clinical isolates of Enterobacteriaceae other than *Escherichia coli*. J. Antimicrob. Chemother..

[52] Chopra I. (2002). New developments in tetracycline antibiotics: Glycylcyclines and tetracycline efflux pump inhibitors. Drug Resist. Updat. Rev. Comment. Antimicrob. Anticancer Chemother..

[53] Farrell D.J., Morrissey I., Bakker S., Morris L., Buckridge S., Felmingham D. (2004). Molecular Epidemiology of Multiresistant *Streptococcus pneumoniae* with Both erm(B)- and mef(A)-Mediated Macrolide Resistance. J. Clin. Microbiol..

[54] Li G., Zhang J., Li C., Guo Q., Jiang Y., Wei J., Qiu Y., Zhao X., Zhao L., Lu J. (2016). Antimycobacterial activity of five efflux pump inhibitors against Mycobacterium tuberculosis clinical isolates. J. Antibiot..

[55] Pieroni M., Machado D., Azzali E., Santos Costa S., Couto I., Costantino G., Viveiros M. (2015). Rational Design and Synthesis of Thioridazine Analogues as Enhancers of the Antituberculosis Therapy. J. Med. Chem..

[56] Bush K. (1988). Beta-lactamase inhibitors from laboratory to clinic. Clin. Microbiol. Rev..

[57] Kapoor G., Saigal S., Elongavan A. (2017). Action and resistance mechanisms of antibiotics: A guide for clinicians. J. Anaesthesiol. Clin. Pharmacol..

[58] Bradford P.A. (2001). Extended-Spectrum -Lactamases in the 21st Century: Characterization, Epidemiology, and Detection of This Important Resistance Threat. Clin. Microbiol. Rev..

[59] Shah A.A., Hasan F., Ahmed S., Hameed A. (2004). Extended-Spectrum β-Lactamases (ESBLs): Characterization, Epidemiology and Detection. Crit. Rev. Microbiol..

[60] Queenan A.M., Bush K. (2007). Carbapenemases: The Versatile -Lactamases. Clin. Microbiol. Rev..

[61] Neu H.C. (1990). β-Lactamases, β-lactamase inhibitors, and skin and skin-structure infections. J. Am. Acad. Dermatol..

[62] Campoli-Richards D.M., Brogden R.N. (1987). Sulbactam/Ampicillin: A Review of its Antibacterial Activity, Pharmacokinetic Properties, and Therapeutic Use. Drugs.

[63] Bryson H.M., Brogden R.N. (1994). Piperacillin/Tazobactam: A Review of its Antibacterial Activity, Pharmacokinetic Properties and Therapeutic Potential. Drugs.

[64] Wise R., Andrews J.M., Bedford K.A. (1978). In vitro study of clavulanic acid in combination with penicillin, amoxycillin, and carbenicillin. Antimicrob. Agents Chemother..

[65] Jacoby G.A., Sutton L. (1989). Pseudomonas cepacia susceptibility to sulbactam. Antimicrob. Agents Chemother..

[66] Flamm R.K., Farrell D.J., Sader H.S., Jones R.N. (2014). Antimicrobial activity of ceftaroline combined with avibactam tested against bacterial organisms isolated from acute bacterial skin and skin structure infections in United States medical centers (2010–2012). Diagn. Microbiol. Infect. Dis..

[67] Biedenbach D.J., Kazmierczak K., Bouchillon S.K., Sahm D.F., Bradford P.A. (2015). In Vitro Activity of Aztreonam-Avibactam against a Global Collection of Gram-Negative Pathogens from 2012 and 2013. Antimicrob. Agents Chemother..

[68] Li H., Estabrook M., Jacoby G.A., Nichols W.W., Testa R.T., Bush K. (2015). In Vitro Susceptibility of Characterized β-Lactamase-Producing Strains Tested with Avibactam Combinations. Antimicrob. Agents Chemother..

[69] Livermore D.M., Mushtaq S., Warner M., Woodford N. (2015). Activity of OP0595/β-lactam combinations against Gram-negative bacteria with extended-spectrum, AmpC and carbapenem-hydrolysing β-lactamases. J. Antimicrob. Chemother..

[70] Hecker S.J., Reddy K.R., Totrov M., Hirst G.C., Lomovskaya O., Griffith D.C., King P., Tsivkovski R., Sun D., Sabet M. (2015). Discovery of a Cyclic Boronic Acid β-Lactamase Inhibitor (RPX7009) with Utility vs Class A Serine Carbapenemases. J. Med. Chem..

[71] Lapuebla A., Abdallah M., Olafisoye O., Cortes C., Urban C., Quale J., Landman D. (2015). Activity of Meropenem Combined with RPX7009, a Novel β-Lactamase Inhibitor, against Gram-Negative Clinical Isolates in New York City. Antimicrob. Agents Chemother..

[72] Vasoo S., Barreto J.N., Tosh P.K. (2015). Emerging Issues in Gram-Negative Bacterial Resistance. Mayo Clin. Proc..

[73] Bonomo R.A. (2017). β-Lactamases: A Focus on Current Challenges. Cold Spring Harb. Perspect. Med..

[74] Delcour A.H. (2009). Outer membrane permeability and antibiotic resistance. Biochim. Biophys. Acta.

[75] Nikaido H. (2003). Molecular Basis of Bacterial Outer Membrane Permeability Revisited. Microbiol. Mol. Biol. Rev..

[76] Zahn M., Bhamidimarri S.P., Baslé A., Winterhalter M., van den Berg B. (2016). Structural Insights into Outer Membrane Permeability of *Acinetobacter baumannii*. Structure.

[77] Li C., Budge L.P., Driscoll C.D., Willardson B.M., Allman G.W., Savage P.B. (1999). Incremental Conversion of Outer-Membrane Permeabilizers into Potent Antibiotics for Gram-Negative Bacteria. J. Am. Chem. Soc..

[78] Kwon D.H., Lu C.-D. (2006). Polyamines Increase Antibiotic Susceptibility in *Pseudomonas aeruginosa*. Antimicrob. Agents Chemother..

[79] Li X.-Z., Nikaido H. (2009). Efflux-Mediated Drug Resistance in Bacteria: An Update. Drugs.

[80] Hurdle J.G., O’Neill A.J., Chopra I., Lee R.E. (2011). Targeting bacterial membrane function: An underexploited mechanism for treating persistent infections. Nat. Rev. Microbiol..

[81] Vooturi S.K., Firestine S.M. (2010). Synthetic Membrane-Targeted Antibiotics. Curr. Med. Chem..

[82] Falagas M.E., Rafailidis P.I., Matthaiou D.K. (2010). Resistance to polymyxins: Mechanisms, frequency and treatment options. Drug Resist. Updat..

[83] Vaara M. (2010). Polymyxins and their novel derivatives. Curr. Opin. Microbiol..

[84] Li Y.-Q., Sun X.-X., Feng J.-L., Mo H.-Z. (2015). Antibacterial activities and membrane permeability actions of glycinin basic peptide against *Escherichia coli*. Innov. Food Sci. Emerg. Technol..

[85] Andrade J.C., Morais Braga M.F.B., Guedes G.M.M., Tintino S.R., Freitas M.A., Quintans L.J., Menezes I.R.A., Coutinho H.D.M. (2017). Menadione (vitamin K) enhances the antibiotic activity of drugs by cell membrane permeabilization mechanism. Saudi. J. Biol. Sci..

[86] Guaní-Guerra E., Santos-Mendoza T., Lugo-Reyes S.O., Terán L.M. (2010). Antimicrobial peptides: General overview and clinical implications in human health and disease. Clin. Immunol..

[87] Ding B., Taotofa U., Orsak T., Chadwell M., Savage P.B. (2004). Synthesis and Characterization of Peptide–Cationic Steroid Antibiotic Conjugates. Org. Lett..

[88] Lai X.-Z., Feng Y., Pollard J., Chin J.N., Rybak M.J., Bucki R., Epand R.F., Epand R.M., Savage P.B. (2008). Ceragenins: Cholic Acid-Based Mimics of Antimicrobial Peptides. Acc. Chem. Res..

[89] Jenssen H., Hamill P., Hancock R.E.W. (2006). Peptide Antimicrobial Agents. Clin. Microbiol. Rev..

[90] Li C., Peters A.S., Meredith E.L., Allman G.W., Savage P.B. (1998). Design and Synthesis of Potent Sensitizers of Gram-Negative Bacteria Based on a Cholic Acid Scaffolding. J. Am. Chem. Soc..

[91] Epand R.M., Epand R.F., Savage P.B. (2008). Ceragenins (Cationic Steroid Compounds), a novel class of antimicrobial agents. Drug News Perspect..

[92] Surel U., Niemirowicz K., Marzec M., Savage P.B., Bucki R. (2014). Ceragenins—A new weapon to fight multi-drug-resistant bacterial infections. Med. Stud..

[93] Dickey S.W., Cheung G.Y.C., Otto M. (2017). Different drugs for bad bugs: Antivirulence strategies in the age of antibiotic resistance. Nat. Rev. Drug Discov..

[94] Cegelski L., Marshall G.R., Eldridge G.R., Hultgren S.J. (2008). The biology and future prospects of antivirulence therapies. Nat. Rev. Microbiol..

[95] Annunziato G., Giovati L., Angeli A., Pavone M., Del Prete S., Pieroni M., Capasso C., Bruno A., Conti S., Magliani W. (2018). Discovering a new class of antifungal agents that selectively inhibits microbial carbonic anhydrases. J. Enzyme Inhib. Med. Chem..

[96] Rasko D.A., Sperandio V. (2010). Anti-virulence strategies to combat bacteria-mediated disease. Nat. Rev. Drug Discov..

[97] Allen R.C., Popat R., Diggle S.P., Brown S.P. (2014). Targeting virulence: Can we make evolution-proof drugs?. Nat. Rev. Microbiol..

[98] Fernebro J. (2011). Fighting bacterial infections—Future treatment options. Drug Resist. Updat..

[99] Brown S.A., Palmer K.L., Whiteley M. (2008). Revisiting the host as a growth medium. Nat. Rev. Microbiol..

[100] Roop R.M., Gaines J.M., Anderson E.S., Caswell C.C., Martin D.W. (2009). Survival of the fittest: How Brucella strains adapt to their intracellular niche in the host. Med. Microbiol. Immunol..

[101] Bhave D.P., Muse W.B., Carroll K.S. (2007). Drug targets in mycobacterial sulfur metabolism. Infect. Disord. Drug Targets.

[102] Becker D., Selbach M., Rollenhagen C., Ballmaier M., Meyer T.F., Mann M., Bumann D. (2006). Robust Salmonella metabolism limits possibilities for new antimicrobials. Nature.

[103] Coates A., Hu Y., Bax R., Page C. (2002). The future challenges facing the development of new antimicrobial drugs. Nat. Rev. Drug Discov..

[104] Coates A.R.M., Hu Y. (2007). Novel approaches to developing new antibiotics for bacterial infections. Br. J. Pharmacol..

[105] Turnbull A.L., Surette M.G. (2008). L-Cysteine is required for induced antibiotic resistance in actively swarming *Salmonella enterica serovar* Typhimurium. Microbiology.

[106] Turnbull A.L., Surette M.G. (2010). Cysteine biosynthesis, oxidative stress and antibiotic resistance in *Salmonella typhimurium*. Res. Microbiol..

[107] Kohanski M.A., Dwyer D.J., Hayete B., Lawrence C.A., Collins J.J. (2007). A Common Mechanism of Cellular Death Induced by Bactericidal Antibiotics. Cell.

[108] Schelle M.W., Bertozzi C.R. (2006). Sulfate Metabolism in Mycobacteria. ChemBioChem.

[109] Mozzarelli A., Bettati S., Campanini B., Salsi E., Raboni S., Singh R., Spyrakis F., Kumar V.P., Cook P.F. (2011). The multifaceted pyridoxal 5′-phosphate-dependent O-acetylserine sulfhydrylase. Biochim. Biophys. Acta.

[110] Kredich N.M. (1971). Regulation of L-cysteine biosynthesis in *Salmonella typhimurium*. I. Effects of growth of varying sulfur sources and O-acetyl-L-serine on gene expression. J. Biol. Chem..

[111] Tai C.H., Nalabolu S.R., Jacobson T.M., Minter D.E., Cook P.F. (1993). Kinetic mechanisms of the A and B isozymes of O-acetylserine sulfhydrylase from *Salmonella typhimurium* LT-2 using the natural and alternate reactants. Biochemistry.

[112] Pearson M.M., Yep A., Smith S.N., Mobley H.L.T. (2011). Transcriptome of Proteus mirabilis in the Murine Urinary Tract: Virulence and Nitrogen Assimilation Gene Expression. Infect. Immun..

[113] Tanous C., Soutourina O., Raynal B., Hullo M.-F., Mervelet P., Gilles A.-M., Noirot P., Danchin A., England P., Martin-Verstraete I. (2008). The CymR Regulator in Complex with the Enzyme CysK Controls Cysteine Metabolism in *Bacillus subtilis*. J. Biol. Chem..

[114] Diner E.J., Beck C.M., Webb J.S., Low D.A., Hayes C.S. (2012). Identification of a target cell permissive factor required for contact-dependent growth inhibition (CDI). Genes Dev..

[115] Pieroni M., Annunziato G., Beato C., Wouters R., Benoni R., Campanini B., Pertinhez T.A., Bettati S., Mozzarelli A., Costantino G. (2016). Rational Design, Synthesis, and Preliminary Structure–Activity Relationships of α-Substituted-2-Phenylcyclopropane Carboxylic Acids as Inhibitors of *Salmonella typhimurium* O-Acetylserine Sulfhydrylase. J. Med. Chem..

[116] Annunziato G., Pieroni M., Benoni R., Campanini B., Pertinhez T.A., Pecchini C., Bruno A., Magalhães J., Bettati S., Franko N. (2016). Cyclopropane-1,2-dicarboxylic acids as new tools for the biophysical investigation of O-acetylserine sulfhydrylases by fluorimetric methods and saturation transfer difference (STD) NMR. J. Enzyme Inhib. Med. Chem..

[117] Amori L., Katkevica S., Bruno A., Campanini B., Felici P., Mozzarelli A., Costantino G. (2012). Design and synthesis of trans-2-substituted-cyclopropane-1-carboxylic acids as the first non-natural small molecule inhibitors of O-acetylserine sulfhydrylase. MedChemComm.

[118] Magalhães J., Annunziato G., Franko N., Pieroni M., Campanini B., Bruno A., Costantino G. (2018). Integration of Enhanced Sampling Methods with Saturation Transfer Difference Experiments to Identify Protein Druggable Pockets. J. Chem. Inf. Model..

[119] Magalhães J., Franko N., Annunziato G., Welch M., Dolan S.K., Bruno A., Mozzarelli A., Armao S., Jirgensons A., Pieroni M. (2018). Discovery of novel fragments inhibiting O-acetylserine sulphhydrylase by combining scaffold hopping and ligand–based drug design. J. Enzyme Inhib. Med. Chem..

[120] Magalhães J., Franko N., Annunziato G., Pieroni M., Benoni R., Nikitjuka A., Mozzarelli A., Bettati S., Karawajczyk A., Jirgensons A. (2019). Refining the structure–activity relationships of 2-phenylcyclopropane carboxylic acids as inhibitors of O-acetylserine sulfhydrylase isoforms. J. Enzyme Inhib. Med. Chem..

[121] Brunner K., Maric S., Reshma R.S., Almqvist H., Seashore-Ludlow B., Gustavsson A.-L., Poyraz Ö., Yogeeswari P., Lundbäck T., Vallin M. (2016). Inhibitors of the Cysteine Synthase CysM with Antibacterial Potency against Dormant *Mycobacterium tuberculosis*. J. Med. Chem..

[122] Franko N., Grammatoglou K., Campanini B., Costantino G., Jirgensons A., Mozzarelli A. (2018). Inhibition of *O*-acetylserine sulfhydrylase by fluoroalanine derivatives. J. Enzyme Inhib. Med. Chem..

[123] Silverman R.B., Abeles R.H. (1976). Inactivation of pyridoxal phosphate dependent enzymes by mono- and polyhaloalanines. Biochemistry.

[124] Alston T.A., Muramatsu H., Ueda T., Bright H.J. (1981). Inactivation of λ-cystathionase by λ-fluorinated amino acids. FEBS Lett..

[125] Azam M.A., Jayaram U. (2016). Inhibitors of alanine racemase enzyme: A review. J. Enzyme Inhib. Med. Chem..

[126] Phillips R.S., Dua R.K. (1992). Indole protects tryptophan indole-lyase, but not tryptophan synthase, from inactivation by trifluoroalanine. Arch. Biochem. Biophys..

[127] Alexeev D., Baxter R.L., Campopiano D.J., Kerbarh O., Sawyer L., Tomczyk N., Watt R., Webster S.P. (2006). Suicide inhibition of α-oxamine synthases: Structures of the covalent adducts of 8-amino-7-oxononanoate synthase with trifluoroalanine. Org. Biomol. Chem..

[128] Tysoe C., Withers S. (2014). Fluorinated Mechanism-Based Inhibitors: Common Themes and Recent Developments. Curr. Top. Med. Chem..

[129] John R.A., Tudball N. (1972). Evidence for Induced Fit of a Pseudo-Substrate of Aspartate Aminotransferase. Eur. J. Biochem..

[130] Passera E., Campanini B., Rossi F., Casazza V., Rizzi M., Pellicciari R., Mozzarelli A. (2011). Human kynurenine aminotransferase II—Reactivity with substrates and inhibitors: Reactivity of kynurenine aminotransferase. FEBS J..

[131] Rutherford S.T., Bassler B.L. (2012). Bacterial Quorum Sensing: Its Role in Virulence and Possibilities for Its Control. Cold Spring Harb. Perspect. Med..

[132] Bassler B.L., Losick R. (2006). Bacterially Speaking. Cell.

[133] Antunes L.C.M., Ferreira R.B.R., Buckner M.M.C., Finlay B.B. (2010). Quorum sensing in bacterial virulence. Microbiology.

[134] Wagner S., Sommer R., Hinsberger S., Lu C., Hartmann R.W., Empting M., Titz A. (2016). Novel Strategies for the Treatment of *Pseudomonas aeruginosa* Infections. J. Med. Chem..

[135] Galloway W.R.J.D., Hodgkinson J.T., Bowden S.D., Welch M., Spring D.R. (2011). Quorum Sensing in Gram-Negative Bacteria: Small-Molecule Modulation of AHL and AI-2 Quorum Sensing Pathways. Chem. Rev..

[136] Geske G.D., O’Neill J.C., Blackwell H.E. (2008). Expanding dialogues: From natural autoinducers to non-natural analogues that modulate quorum sensing in Gram-negative bacteria. Chem. Soc. Rev..

[137] Köhler T., Perron G.G., Buckling A., van Delden C. (2010). Quorum Sensing Inhibition Selects for Virulence and Cooperation in *Pseudomonas aeruginosa*. PLoS Pathog..

[138] Starkey M., Lepine F., Maura D., Bandyopadhaya A., Lesic B., He J., Kitao T., Righi V., Milot S., Tzika A. (2014). Identification of Anti-virulence Compounds That Disrupt Quorum-Sensing Regulated Acute and Persistent Pathogenicity. PLoS Pathog..

[139] Ilangovan A., Fletcher M., Rampioni G., Pustelny C., Rumbaugh K., Heeb S., Cámara M., Truman A., Chhabra S.R., Emsley J. (2013). Structural Basis for Native Agonist and Synthetic Inhibitor Recognition by the *Pseudomonas aeruginosa* Quorum Sensing Regulator PqsR (MvfR). PLoS Pathog..

[140] Mistry S., Roy S., Maitra N.J., Kundu B., Chanda A., Datta S., Joy M. (2016). A novel, multi-barrier, drug eluting calcium sulfate/biphasic calcium phosphate biodegradable composite bone cement for treatment of experimental MRSA osteomyelitis in rabbit model. J. Control. Release.

[141] De la Fuente-Núñez C., Reffuveille F., Haney E.F., Straus S.K., Hancock R.E.W. (2014). Broad-Spectrum Anti-biofilm Peptide That Targets a Cellular Stress Response. PLoS Pathog..

[142] Reffuveille F., de la Fuente-Núñez C., Mansour S., Hancock R.E.W. (2014). A Broad-Spectrum Antibiofilm Peptide Enhances Antibiotic Action against Bacterial Biofilms. Antimicrob. Agents Chemother..

[143] Koo H., Allan R.N., Howlin R.P., Stoodley P., Hall-Stoodley L. (2017). Targeting microbial biofilms: Current and prospective therapeutic strategies. Nat. Rev. Microbiol..

[144] De la Fuente-Núñez C., Reffuveille F., Fernández L., Hancock R.E. (2013). Bacterial biofilm development as a multicellular adaptation: Antibiotic resistance and new therapeutic strategies. Curr. Opin. Microbiol..

[145] Flemming H.-C., Wingender J., Szewzyk U., Steinberg P., Rice S.A., Kjelleberg S. (2016). Biofilms: An emergent form of bacterial life. Nat. Rev. Microbiol..

[146] Van Acker H., Van Dijck P., Coenye T. (2014). Molecular mechanisms of antimicrobial tolerance and resistance in bacterial and fungal biofilms. Trends Microbiol..

[147] Lebeaux D., Ghigo J.-M., Beloin C. (2014). Biofilm-Related Infections: Bridging the Gap between Clinical Management and Fundamental Aspects of Recalcitrance toward Antibiotics. Microbiol. Mol. Biol. Rev..

